# Supplementary material for: HIVprotI: an integrated web based platform for prediction and design of HIV proteins inhibitors
Source: J Cheminform. 2018 Mar 9;10:12. doi: 10.1186/s13321-018-0266-y (PMC5845081; doi:10.1186/s13321-018-0266-y)
Supplement: Supplementary file 2 — Additional file 2. Source code of HIVProtI web server. [file 13321_2018_266_MOESM2_ESM.zip › HIVprotI_Source-code/hivprotI/weka-3-6-6/doc/weka/gui/GenericObjectEditor.html]

GenericObjectEditor


---


|  |  |  |
| --- | --- | --- |
| **Overview** \| **Package** \| **Class** \| **Tree** \| **Deprecated** \| **Index** \| **Help** \| **Weka's home** | |  |
| **PREV CLASS**   **NEXT CLASS** | **FRAMES**    **NO FRAMES**     **All Classes** |  |
| SUMMARY: NESTED \| FIELD \| CONSTR \| METHOD | DETAIL: FIELD \| CONSTR \| METHOD |  |


---


## weka.gui Class GenericObjectEditor

```
java.lang.Object
  weka.gui.GenericObjectEditor
```

**All Implemented Interfaces:**: java.beans.PropertyEditor, CustomPanelSupplier

---

``` public class GenericObjectEditor extends java.lang.Object implements java.beans.PropertyEditor, CustomPanelSupplier ```

A PropertyEditor for objects. It can be used either in a static or a dynamic
way.   
  
In the **static** way (`USE_DYNAMIC` is `false`) the
objects have been defined as editable in the GenericObjectEditor
configuration file, which lists possible values that can be selected from,
and themselves configured. The configuration file is called
"GenericObjectEditor.props" and may live in either the location given by
"user.home" or the current directory (this last will take precedence), and a
default properties file is read from the Weka distribution. For speed, the
properties file is read only once when the class is first loaded -- this may
need to be changed if we ever end up running in a Java OS ;-).   
  
If it is used in a **dynamic** way (the `UseDynamic` property
of the GenericPropertiesCreator props file is set to `true`)
then the classes to list are discovered by the
`GenericPropertiesCreator` class (it checks the complete classpath).

**Version:**
:   $Revision: 7059 $

**Author:**
:   Len Trigg (trigg@cs.waikato.ac.nz), Xin Xu (xx5@cs.waikato.ac.nz), Richard Kirkby (rkirkby@cs.waikato.ac.nz), FracPete (fracpete at waikato dot ac dot nz)

**See Also:**: `GenericPropertiesCreator`, `GenericPropertiesCreator.useDynamic()`, `GenericPropertiesCreator.CREATOR_FILE`, `ClassDiscovery`

---

| **Nested Class Summary** | |
| --- | --- |
| `class` | `GenericObjectEditor.CapabilitiesFilterDialog`             A dialog for selecting Capabilities to look for in the GOE tree. |
| `class` | `GenericObjectEditor.GOEPanel`             Handles the GUI side of editing values. |
| `class` | `GenericObjectEditor.GOETreeNode`             A specialized TreeNode for supporting filtering via Capabilities. |
| `class` | `GenericObjectEditor.JTreePopupMenu`             Creates a popup menu containing a tree that is aware of the screen dimensions. |


| **Field Summary** | |
| --- | --- |
| `static java.lang.String` | `GUIEDITORS_PROPERTY_FILE`             the properties files containing the class/editor mappings. |


**Constructor Summary**

`GenericObjectEditor()`
  
          Default constructor.

`GenericObjectEditor(boolean canChangeClassInDialog)`
  
          Constructor that allows specifying whether it is possible
to change the class within the editor dialog.


| **Method Summary** | |
| --- | --- |
| `void` | `addPropertyChangeListener(java.beans.PropertyChangeListener l)`             Adds a PropertyChangeListener who will be notified of value changes. |
| `java.lang.String` | `getAsText()`             Returns null as we don't support getting/setting values as text. |
| `java.lang.Object` | `getBackup()`             Returns the backup object (may be null if there is no backup. |
| `boolean` | `getCanChangeClassInDialog()`             Returns whether the user can change the class in the dialog. |
| `Capabilities` | `getCapabilitiesFilter()`             Returns the current Capabilities filter, can be null. |
| `javax.swing.JPopupMenu` | `getChooseClassPopupMenu()`             Returns a popup menu that allows the user to change the class of object. |
| `static java.util.Vector<java.lang.String>` | `getClassnames(java.lang.String property)`             Returns the available classnames for a certain property in the props file. |
| `java.awt.Component` | `getCustomEditor()`             Returns the array editing component. |
| `javax.swing.JPanel` | `getCustomPanel()`             Gets the custom panel used for editing the object. |
| `java.lang.String` | `getJavaInitializationString()`             Supposedly returns an initialization string to create a Object identical to the current one, including it's state, but this doesn't appear possible given that the initialization string isn't supposed to contain multiple statements. |
| `java.lang.String[]` | `getTags()`             Returns null as we don't support getting values as tags. |
| `java.lang.Object` | `getValue()`             Gets the current Object. |
| `boolean` | `isPaintable()`             Returns true to indicate that we can paint a representation of the Object. |
| `static void` | `main(java.lang.String[] args)`             Tests out the Object editor from the command line. |
| `static java.lang.Object` | `makeCopy(java.lang.Object source)`             Makes a copy of an object using serialization. |
| `void` | `paintValue(java.awt.Graphics gfx, java.awt.Rectangle box)`             Paints a representation of the current Object. |
| `static void` | `registerEditors()`             registers all the editors in Weka. |
| `void` | `removeCapabilitiesFilter()`             Removes the current Capabilities filter. |
| `void` | `removePropertyChangeListener(java.beans.PropertyChangeListener l)`             Removes a PropertyChangeListener. |
| `void` | `setAsText(java.lang.String text)`             Returns null as we don't support getting/setting values as text. |
| `void` | `setCanChangeClassInDialog(boolean value)`             Sets whether the user can change the class in the dialog. |
| `void` | `setCapabilitiesFilter(Capabilities value)`             Sets the capabilities to use for filtering. |
| `void` | `setClassType(java.lang.Class type)`             Sets the class of values that can be edited. |
| `void` | `setDefaultValue()`             Sets the current object to be the default, taken as the first item in the chooser. |
| `void` | `setEnabled(boolean newVal)`             Sets whether the editor is "enabled", meaning that the current values will be painted. |
| `void` | `setValue(java.lang.Object o)`             Sets the current Object. |
| `static java.util.Hashtable` | `sortClassesByRoot(java.lang.String classes)`             parses the given string of classes separated by ", " and returns the a hashtable with as many entries as there are different root elements in the class names (the key is the root element). |
| `boolean` | `supportsCustomEditor()`             Returns true because we do support a custom editor. |

**Methods inherited from class java.lang.Object**

`equals, getClass, hashCode, notify, notifyAll, toString, wait, wait, wait`

**Field Detail**

### GUIEDITORS\_PROPERTY\_FILE

```
public static final java.lang.String GUIEDITORS_PROPERTY_FILE
```

:   the properties files containing the class/editor mappings.

    **See Also:**: Constant Field Values


**Constructor Detail**

### GenericObjectEditor

```
public GenericObjectEditor()
```

:   Default constructor.

---


### GenericObjectEditor

```
public GenericObjectEditor(boolean canChangeClassInDialog)
```

:   Constructor that allows specifying whether it is possible
    to change the class within the editor dialog.

    **Parameters:**: `canChangeClassInDialog` - whether the user can change the class


**Method Detail**

### registerEditors

```
public static void registerEditors()
```

:   registers all the editors in Weka.

---


### setCanChangeClassInDialog

```
public void setCanChangeClassInDialog(boolean value)
```

:   Sets whether the user can change the class in the dialog.

    :   **Parameters:**: `value` - if true then the user can change the class

---


### getCanChangeClassInDialog

```
public boolean getCanChangeClassInDialog()
```

:   Returns whether the user can change the class in the dialog.

    :   **Returns:**: true if the user can change the class

---


### getBackup

```
public java.lang.Object getBackup()
```

:   Returns the backup object (may be null if there is no
    backup.

    :   **Returns:**: the backup object

---


### sortClassesByRoot

```
public static java.util.Hashtable sortClassesByRoot(java.lang.String classes)
```

:   parses the given string of classes separated by ", " and returns the
    a hashtable with as many entries as there are different root elements in
    the class names (the key is the root element). E.g. if there's only
    "weka." as the prefix for all classes the a hashtable of size 1 is returned.
    if NULL is the input, then NULL is also returned.

    :   **Parameters:**: `classes` - the classnames to work on **Returns:**: for each distinct root element in the classnames, one entry in the hashtable (with the root element as key)

---


### setEnabled

```
public void setEnabled(boolean newVal)
```

:   Sets whether the editor is "enabled", meaning that the current
    values will be painted.

    :   **Parameters:**: `newVal` - a value of type 'boolean'

---


### setClassType

```
public void setClassType(java.lang.Class type)
```

:   Sets the class of values that can be edited.

    :   **Parameters:**: `type` - a value of type 'Class'

---


### setDefaultValue

```
public void setDefaultValue()
```

:   Sets the current object to be the default, taken as the first item in
    the chooser.

---


### setValue

```
public void setValue(java.lang.Object o)
```

:   Sets the current Object. If the Object is in the
    Object chooser, this becomes the selected item (and added
    to the chooser if necessary).

    :   **Specified by:**: `setValue` in interface `java.beans.PropertyEditor`
    :   **Parameters:**: `o` - an object that must be a Object.

---


### getValue

```
public java.lang.Object getValue()
```

:   Gets the current Object.

    :   **Specified by:**: `getValue` in interface `java.beans.PropertyEditor`
    :   **Returns:**: the current Object

---


### getJavaInitializationString

```
public java.lang.String getJavaInitializationString()
```

:   Supposedly returns an initialization string to create a Object
    identical to the current one, including it's state, but this doesn't
    appear possible given that the initialization string isn't supposed to
    contain multiple statements.

    :   **Specified by:**: `getJavaInitializationString` in interface `java.beans.PropertyEditor`
    :   **Returns:**: the java source code initialisation string

---


### isPaintable

```
public boolean isPaintable()
```

:   Returns true to indicate that we can paint a representation of the
    Object.

    :   **Specified by:**: `isPaintable` in interface `java.beans.PropertyEditor`
    :   **Returns:**: true

---


### paintValue

```
public void paintValue(java.awt.Graphics gfx,
                       java.awt.Rectangle box)
```

:   Paints a representation of the current Object.

    :   **Specified by:**: `paintValue` in interface `java.beans.PropertyEditor`
    :   **Parameters:**: `gfx` - the graphics context to use: `box` - the area we are allowed to paint into

---


### getAsText

```
public java.lang.String getAsText()
```

:   Returns null as we don't support getting/setting values as text.

    :   **Specified by:**: `getAsText` in interface `java.beans.PropertyEditor`
    :   **Returns:**: null

---


### setAsText

```
public void setAsText(java.lang.String text)
```

:   Returns null as we don't support getting/setting values as text.

    :   **Specified by:**: `setAsText` in interface `java.beans.PropertyEditor`
    :   **Parameters:**: `text` - the text value **Throws:**: `java.lang.IllegalArgumentException` - as we don't support getting/setting values as text.

---


### getTags

```
public java.lang.String[] getTags()
```

:   Returns null as we don't support getting values as tags.

    :   **Specified by:**: `getTags` in interface `java.beans.PropertyEditor`
    :   **Returns:**: null

---


### supportsCustomEditor

```
public boolean supportsCustomEditor()
```

:   Returns true because we do support a custom editor.

    :   **Specified by:**: `supportsCustomEditor` in interface `java.beans.PropertyEditor`
    :   **Returns:**: true

---


### getCustomEditor

```
public java.awt.Component getCustomEditor()
```

:   Returns the array editing component.

    :   **Specified by:**: `getCustomEditor` in interface `java.beans.PropertyEditor`
    :   **Returns:**: a value of type 'java.awt.Component'

---


### addPropertyChangeListener

```
public void addPropertyChangeListener(java.beans.PropertyChangeListener l)
```

:   Adds a PropertyChangeListener who will be notified of value changes.

    :   **Specified by:**: `addPropertyChangeListener` in interface `java.beans.PropertyEditor`
    :   **Parameters:**: `l` - a value of type 'PropertyChangeListener'

---


### removePropertyChangeListener

```
public void removePropertyChangeListener(java.beans.PropertyChangeListener l)
```

:   Removes a PropertyChangeListener.

    :   **Specified by:**: `removePropertyChangeListener` in interface `java.beans.PropertyEditor`
    :   **Parameters:**: `l` - a value of type 'PropertyChangeListener'

---


### getCustomPanel

```
public javax.swing.JPanel getCustomPanel()
```

:   Gets the custom panel used for editing the object.

    :   **Specified by:**: `getCustomPanel` in interface `CustomPanelSupplier`
    :   **Returns:**: the panel

---


### getChooseClassPopupMenu

```
public javax.swing.JPopupMenu getChooseClassPopupMenu()
```

:   Returns a popup menu that allows the user to change
    the class of object.

    :   **Returns:**: a JPopupMenu that when shown will let the user choose the class

---


### setCapabilitiesFilter

```
public void setCapabilitiesFilter(Capabilities value)
```

:   Sets the capabilities to use for filtering.

    :   **Parameters:**: `value` - the object to get the filter capabilities from

---


### getCapabilitiesFilter

```
public Capabilities getCapabilitiesFilter()
```

:   Returns the current Capabilities filter, can be null.

    :   **Returns:**: the current Capabiliities used for filtering

---


### removeCapabilitiesFilter

```
public void removeCapabilitiesFilter()
```

:   Removes the current Capabilities filter.

---


### makeCopy

```
public static java.lang.Object makeCopy(java.lang.Object source)
                                 throws java.lang.Exception
```

:   Makes a copy of an object using serialization.

    :   **Parameters:**: `source` - the object to copy **Returns:**: a copy of the source object **Throws:**: `java.lang.Exception` - if the copy fails

---


### getClassnames

```
public static java.util.Vector<java.lang.String> getClassnames(java.lang.String property)
```

:   Returns the available classnames for a certain property in the
    props file.

    :   **Parameters:**: `property` - the property to get the classnames for **Returns:**: the classnames

---


### main

```
public static void main(java.lang.String[] args)
```

:   Tests out the Object editor from the command line.

    :   **Parameters:**: `args` - may contain the class name of a Object to edit


---


|  |  |  |
| --- | --- | --- |
| **Overview** \| **Package** \| **Class** \| **Tree** \| **Deprecated** \| **Index** \| **Help** \| **Weka's home** | |  |
| **PREV CLASS**   **NEXT CLASS** | **FRAMES**    **NO FRAMES**     **All Classes** |  |
| SUMMARY: NESTED \| FIELD \| CONSTR \| METHOD | DETAIL: FIELD \| CONSTR \| METHOD |  |


---
